# The Influence of Thickness and Spectral Properties of Green Color-Emitting Polymer Thin Films on Their Implementation in Wearable PLED Applications

**DOI:** 10.3390/nano14191608

**Published:** 2024-10-07

**Authors:** Kyparisis Papadopoulos, Despoina Tselekidou, Alexandros Zachariadis, Argiris Laskarakis, Stergios Logothetidis, Maria Gioti

**Affiliations:** 1 Nanotechnology Laboratory LTFN, Department of Physics, Aristotle University of Thessaloniki, 54124 Thessaloniki, Greece; detselek@physics.auth.gr (D.T.); alzac@physics.auth.gr (A.Z.); alask@physics.auth.gr (A.L.); logot@auth.gr (S.L.); 2 Organic Electronic Technologies P.C. (OET), 20th km Thessaloniki—Tagarades, 57001 Thermi, Greece

**Keywords:** PLED, flexible electronics, rhodamine 6G, optical sensor, solution process

## Abstract

A systematic investigation of optical, electrochemical, photophysical, and electrooptical properties of printable green color-emitting polymer (poly(9,9-dioctylfluorene-alt-bithiophene)) (F8T2) and spiro-copolymer (SPG-01T) was conducted to explore their potentiality as an emissive layer for wearable polymer light-emitting diode (PLED) applications. We compared the two photoactive polymers in terms of their spectral characteristics and color purity, as these are the most critical factors for wearable lighting sources and optical sensors. Low-cost, solution-based methods and facile architecture were applied to produce rigid and flexible light-emitting devices with high luminance efficiencies. Emission bandwidths, color coordinates, operational characteristics, and luminance were also derived to evaluate the device’s stability. The tuning of emission’s spectral features by layer thickness variation was realized and was correlated with the interplay between H-aggregates and J-aggregates formations for both conjugated polymers. Finally, we applied the functional green light-emitting PLED devices based on the two studied materials for the detection of Rhodamine 6G. It was determined that the optical detection of the R6G photoluminescence is heavily influenced by the emission spectrum characteristics of the PLED and changes in the thickness of the active layer.

## 1. Introduction

In recent years, organic light-emitting diodes (OLEDs) have been considered the next generation of lighting sources. One of their most attractive applications is the implementation of OLEDs in wearables due to their ability for fabrication on flexible substrates, giving them the advantage of conforming to curved surfaces such as the human body [1,2,3]. Wearable optical sensors, based on absorbance, reflectance, luminescence (fluorescence or phosphorescence), and light scattering principles, have become increasingly popular in recent years due to their improved reliability and suitability for non-invasive investigations [4,5,6,7].

Polymer-based OLEDs (PLEDs) can be produced by low-cost, wet chemistry techniques such as spin coating, slot-die coating, or inkjet printing [8,9,10]. Such processes are compatible with plastic surfaces and can initiate the scaling up of PLED-based optical sensors and lighting devices in roll-to-roll (R2R) production lines.

The operational characteristics of PLEDs are another important issue that should be addressed for their incorporation into lighting and sensing applications. Performance with relatively low current density and high luminance efficiency can improve the signal-to-noise ratio (SNR) of the sensor’s light detector and increase the overall lifetime of the device [11].

The color purity and stability of light-emitting devices are critical factors to consider when designing optical sensors as they directly impact the accuracy and reliability of sensor measurements. These factors also play a crucial role in determining the emission quality when the devices are intended for lighting applications. In PLEDs, both characteristics are closely linked to the structural and optical properties of the π-conjugated polymers employed in the devices [12,13]. Greater charge transfer interactions within the polymer system result in broader emission spectra, leading to reduced monochromaticity. Nevertheless, broadband spectrum is not a limiting factor as it is beneficial when comparing signals across multiple wavelengths, such as in the measurement of blood oxygenation levels and heart rate [9]. Conversely, narrow spectra are more desirable when focusing on specific excitation targets [14]. On the other hand, color stability is not negotiable. The International Telecommunication Union (ITU) has proposed several color gamut standards. The most widespread system is BT709 where CIE coordinates for the red, green, and blue colors are (0.64, 0.33), (0.30, 0.60), and (0.15, 0.06), respectively [15]. So, these values remaining stable during different luminance levels and environmental conditions is considered favorable for wearable PLED-based applications.

Implementation of green color-emitting polymers as active layers in PLEDs, except for the commonly accepted benefits of solution processability, flexibility, stability, and tunability of optoelectronic properties [16,17], can offer the capability to detect and monitor biomolecules or chemical processes. Rhodamine 6G (R6G) is a fluorescent dye commonly utilized in a variety of applications such as laser technology, fluorescence microscopy, and fluid dynamics research as a fluorescent tracer. Because of its high fluorescence, it is perfect for optical sensing applications where variations in wavelength or fluorescence intensity can reveal the presence or the concentration of particular analytes [18,19]. Many research groups have already published studies concerning the integration of green color OLEDs in PL-based sensors or in wearable pulse oximeters [14,20,21]. However, complex architectures are developed containing microcavities or optical filters to improve color purity whereas, in order to achieve broadband emission, multilayered stacks are fabricated with blended systems as emissive layers that may undergo phase separation. Nevertheless, there is still a need for further exploration of structural and photophysical appropriate printable green-emitting polymers in order to widen the utilization of PLEDs beyond their current limits.

In this work, we studied two commercially available green-light photoactive polymers for their capability to be implemented on simple, structured, and printed solution-processed green color PLEDs: poly(9,9-dioctylfluorene-alt-bithiophene) (F8T2) and green spiro-copolymer (SPG-01T). Emission bandwidths and color coordinates were derived for their potential application in wearable biosensing technology and lighting. The printability of the polymer films on flexible substrates was tested using the slot-die coating technique. Also, the tuning of their electro-optical properties by the film’s thickness variation was investigated and the optical performance was correlated with the operational characteristics of the fabricated PLED devices. Finally, we present preliminary results concerning the application of the green color PLEDs as actuators for the detection of R6G PL.

## 2. Materials and Methods

### 2.1. Ink Formulation

For the hole transport layer (HTL), poly-3,4-ethylene dioxythiophene: poly-styrene sulfonate, PEDOT: PSS, (PVP Al 4083) purchased from Heraeus was used and mixed with ethanol at a ratio of 2:1. The solution was agitated for a duration of 3 h and then passed through a PVDF syringe filter with a pore size of 0.45 μm. The two photoactive materials, poly(9,9-dioctylfluorene-alt-bithiophene) (F8T2) (Mn > 20,000) and green light-emitting spiro-copolymer (SPG-01T) (Mn > 100,000), as well as Rhodamine (R6G) were obtained from Sigma-Aldrich Chemie GmbH (St. Louis, MO, USA). A solution of 6.94 mg/mL of F8T2 and SPG-01T was dissolved in toluene and subjected to heating and stirring overnight. For R6G, a 5 × 10^−4^ M solution was prepared by dissolving it in 3 mL of deionized water.

### 2.2. PLED Fabrication

**Scheme 1:**
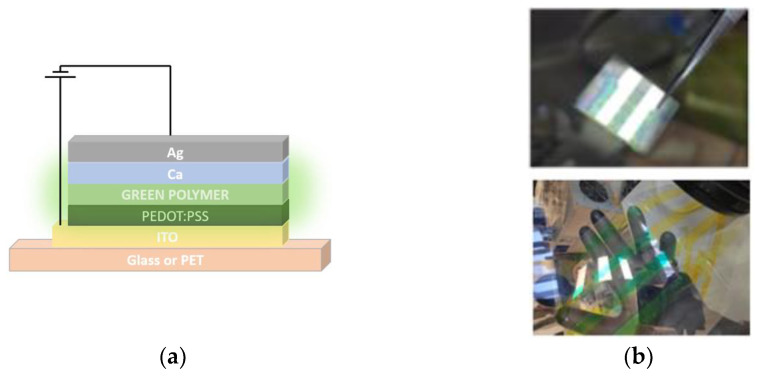
(**a**) The architecture of the fabricated devices, (**b**) spin-coated PLED (**up**) and slot-die-coated stripes (**down**).

The selected architecture for the rigid and flexible devices is presented in Scheme 1a. The spin-coated PLEDs were prepared on pre-cleaned indium-tin oxide (ITO) glass substrates (by Ossila, Sheffield, UK) which were initially cleaned by sequential ultrasonication in deionized water, acetone, and ethanol for 10 min and then dried by nitrogen. Afterwards, an oxygen plasma treatment at 40 W for 3 min was performed inside the glovebox, and the PEDOT: PSS solution was spin coated at 4500 rpm for 60 s on the pretreated ITO substrates. Thermal annealing at 120 °C for 5 min was applied to the aqueous PEDOT: PSS layers for the removal of residual water. For the next step, emissive layers (EML) based on F8T2 and SPG-01T were produced by spin coating at 1000 rpm, 2000 rpm, and 3000 rpm for 60 s. The fabrication process of the PLED devices was finalized by performing the sequential thermal evaporation of 6 nm of Ca as the Electron Injection Layer (EIL) and 120 nm of Ag as the cathode electrode under a base pressure of approximately 10^−6^ mbar. To create the desired pixel pattern as depicted in the upper picture of Scheme 1b, with dimensions of 0.04 cm^2^ for each pixel, appropriate shadow masks were employed during the deposition.

The fabrication of the flexible devices was performed at a FOM Technologies Mini Roll Coater (MRC) which mimics R2R production procedure. Firstly, a 1 m long PET substrate patterned with ITO was mounted on the roller using heat-stable tape. Then, a layer of 40 nm PEDOT: PSS was slot-die coated onto the substrate. This was performed by applying the coating in the form of 13 mm wide stripes, displayed in the lower picture of Scheme 1b. The distance between the nozzle-substrate and the flow rate of the ink was set at 120 μm and 0.045 mL/min, respectively. The coating process took place at a roll temperature of 45 °C with a web speed of 0.5 m/min. Then, the substrate was transferred to a vacuum oven for thermal annealing at 120 °C for 5 min. Afterwards, the substrate was attached again to the roller and the active layer consisting of the solution of each green polymer was coated on top of the HTL with an offset of 1 mm to ensure electrical contact with the anode electrode. Τhe nozzle-substrate distance and the web speed values remained constant whereas the roll temperature increased at 50 °C and different flow rates were applied to the green polymer-based ink in order to achieve thickness variation. Subsequently, the printed stripes were cut into smaller ones in order to be appropriately adapted to the thermal evaporator’s shadow mask and produce pixels of 1 × 1.2 cm^2^. Finally, Ca and then Ag were evaporated with the same parameters mentioned above.

### 2.3. Thin Film and Device Characterization

The optical properties of the fabricated thin films were investigated by spectroscopic ellipsometry (SE). The experimental setup used was a phase-modulated ellipsometer (UVISEL Jobin Yvon, Horiba Europe Research Center, Palaiseau, France). The SE measurements were conducted from the near IR to far UV spectral region 1.5–6.5 eV with a step of 20 meV at a 70° angle of incidence. The produced data, taking into consideration all the fitting parameters, were fitted to model-generated data using the Levenberg-Marquardt algorithm.

Photophysical characteristics were studied via photoluminescence (PL) measurements, using the Hamamatsu Absolute PL Quantum Yield system (C9920-02) (Jokocho, Hamamatsu, Japan). The absorbance measurements were performed using the FR UV/VIS model of ThetaMetrisis covering the spectral range of 300–700 nm using a deuterium and halogen light source.

Cyclic voltammetry (CV) is a frequently used method for understanding the electrochemical behavior of materials. CV was performed on an Autolab Potentionstat (Metrohm, Herisau, Switzerland). The measurements were conducted in a system with a three-electrode setup where a fluorine-doped indium-tin oxide (FTO) glass substrate was used as the working electrode while an Ag/AgCl (KCl_(Sat)_) and a Pt wire were applied as the working and auxiliary electrode, respectively. The films of each polymer were drop casted on FTO-coated glass slides and were further annealed at 80 °C for 5 min. Tetrabutylammonium hexafluorophosphate (TBAPF_6_) 0.1 M dissolved in acetonitrile (CH_3_CN) was used as a supporting electrolyte whereas the pair ferrocene/ferrocenium was implemented for calibration. The experiments were conducted in the range of 0–2.5 V with a step of 0.1 V/s.

The produced PLED devices were examined in a glovebox without encapsulation by the Hamamatsu external quantum efficiency system C9920-12 (Jokocho, Hamamatsu, Japan), which measures the brightness and light distribution of the emitting diode. The current density-voltage (J-V) characteristics of the PLEDs were tested by applying a Keithley 2420 source measurement unit interfaced with a PC.

## 3. Results

### 3.1. Photophysical and Electrochemical Characterization of Thin Films

**Figure 1:**
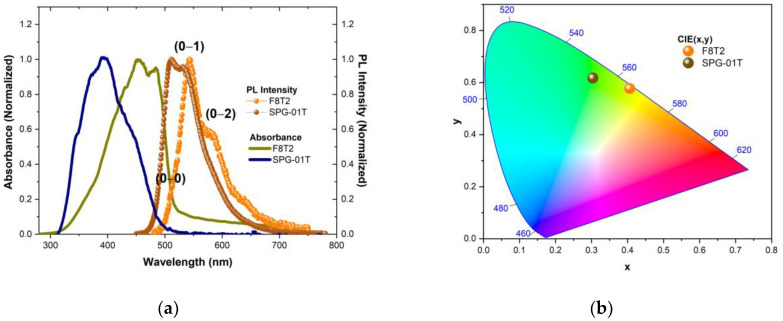
(**a**) The UV-Vis absorption and photoluminescence spectra of the F8T2 and SPG-01T thin films, (**b**) the respective chromaticity coordinates.

In Figure 1a the absorbance and PL spectra of the F8T2 and SPG-01T films are plotted together. For F8T2, there are two characteristic absorption peaks at 484 nm and 453 nm, which can be correlated with π-π* electronic transitions between bonding and antibonding molecular orbitals [22]. Concerning the F8T2 polymer’s emission spectrum, it has an intensively vibrational character with a sharp peak at 542 nm and a shoulder peak at 581 nm assigned to (0–1) and (0–2), respectively, whereas the peak at 510 nm, related to the (0–0) relaxation [23], has a weak contribution to the emission profile. For the SPG-01T, there is a significant contribution of the (0–0) peaks at 442 nm in the absorbance spectrum and 509 nm in the PL spectrum. Additionally, intense (0–1) peaks are observed at 397 nm and 537 nm in the respective spectra. In Figure 1b, the CIE coordinates of the two green color polymers are displayed. F8T2 exhibits a yellow-green emission whereas SPG-01T’s color coordinates are in the deep green region. The photophysical properties of the two polymers are summarized in Table 1.

CV was conducted to investigate the electrochemical properties and to calculate the highest occupied molecular orbital (HOMO) and lowest unoccupied molecular orbital (LUMO) levels of F8T2 and SPG-01T to define the energy differences between the films based on the green color polymers and the other layers of the PLED’s architecture. According to this method, when a specific electrical potential is reached (E_onset_), the electrode’s surface undergoes oxidation, leading to the occurrence of anodic currents. By knowing the standard redox potential of the ferrocene/ferrocenium couple ($E_{\frac{FC}{FC+}}$) in the supporting electrolyte of CV and the Fermi level of ferrocene being 4.8 eV, it becomes possible to estimate the energy level of the HOMO (E_HOMO_), according to the relation:

(1) $$E_{HOMO}=e\left(E_{onset}-E_{\frac{FC}{FC+}}\right)-4.8\;eV$$

Similarly, the LUMO energy level (E_LUMO_) can be calculated by subtracting the optical bandgap energy (E_g_^Opt^), obtained from the absorption spectrum using the equation:

(2) $$E_{g}^{Opt}=\frac{1240}{\lambda_{onset}}\;eV$$

where λ_onset_ is the value of the onset absorption wavelength derived, for each green color emitting polymer, by Figure 1a. In other words, the equation used to determine the LUMO level is:

(3) $$E_{LUMO}=\left(E_{HOMO}+E_{g}^{Opt}\right)\;eV$$

**Figure 2:**
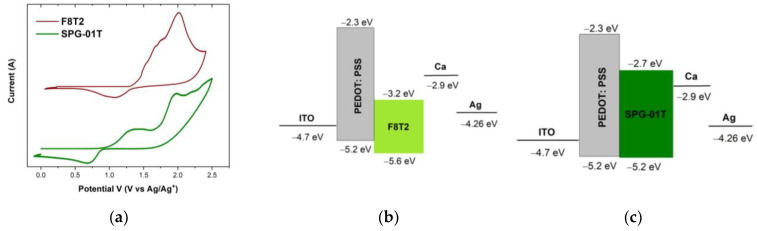
(**a**) Cyclic voltammetry of both green color-emitting polymers. Energy level diagram of proposed architecture based on (**b**) F8T2 and (**c**) SPG-01T as emitting material.

The voltammograms for both polymers are presented in Figure 2a and the estimated values are listed in Table 1 [24,25].

The HOMO energy of F8T2 at −5.6 eV is higher than the corresponding energy of SPG-01T at −5.2 eV. On the other hand, the opposite situation takes place considering the LUMO level of the two polymers is −3.2 eV for F8T2 film whereas it is −2.71 eV for SPG-01T. Observing the energy diagrams of the fabricated PLEDs in Figure 2b,c, there is an almost perfect alignment between the HOMO levels of SPG-01T and PEDOT: PSS and an energy difference of 0.4 eV between the emissive material and Ca. In the case of F8T2, the LUMO level is lower by 0.3 eV than the work function of Ca whereas there is a 0.4 eV energy step between the HTL and the HOMO level. Consequently, we can assume that, concerning the F8T2-based PLEDs, they are governed by excessive electron injection while in the SPG-01T-based devices, the holes are injected more efficiently, having a more balanced recombination rate due to the low energy barrier of electrons [26,27].

### 3.2. Dependence of Optical Characteristics and Morphological Features of F8T2 and SPG-01T on Thickness

Spectroscopic ellipsometry (SE) is a powerful, surface-sensitive technique used to measure the dielectric function of materials to determine their optical properties. When light interacts with a material, its polarization state can be altered due to various optical phenomena such as reflection, refraction, absorption, and scattering. SE exploits these changes to extract information about the material’s opto-electronic response.

When dealing with thin films in single or multilayer structures, SE measures the pseudodielectric function $<\varepsilon \left(Ε\right)>=<\varepsilon_{1}\left(Ε\right)>+<\varepsilon_{2}\left(Ε\right)>$, which takes into account both the dielectric response and the films’ thicknesses. Analyzing multilayer samples is more complex, as the layer sequence must be included in the optical model for simulation and fitting. By employing the appropriate modeling procedures, it is possible to calculate the thickness of layers with sub-nanometer resolution. Furthermore, the spectral dependence of the dielectric function, absorption coefficient, and other numerical optical parameters such as the fundamental band gap, absorption energies, and bandwidths can be derived as they are incorporated in the dispersion equations that describe the dielectric response of the films.

The modified Tauc–Lorentz (TL) dispersion oscillator model was used in conjunction with the five-phase geometrical model Air/Emitting Film/PEDOT:PSS/ITO/Glass or PET substrate in the spin-coated (SC) and slot-die-coated (SD) stacks, respectively. This modeling procedure has been shown to contribute successfully to the analysis of amorphous organic semiconductors [28,29,30,31]. A comprehensive description of the model and the SE analysis can be found elsewhere [32].

**Figure 3:**
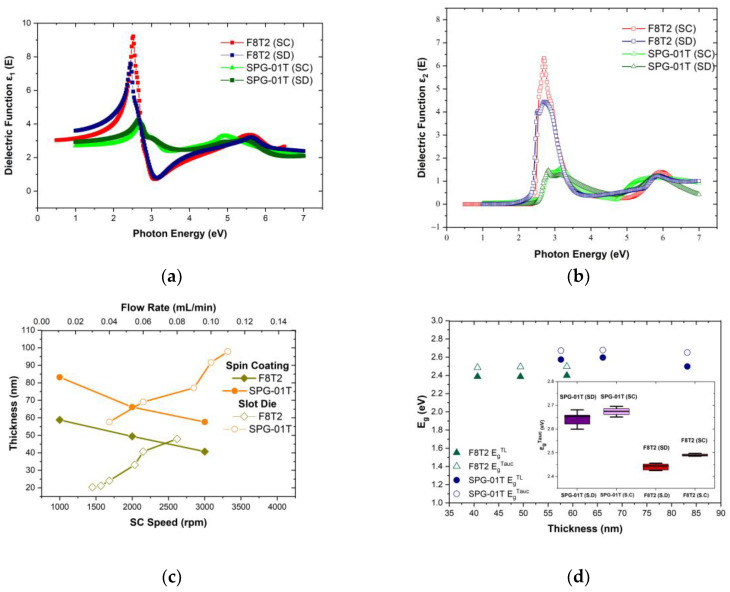
The real ε_1_(Ε) (**a**) and imaginary part ε_2_(Ε) (**b**) of the dielectric function ε(Ε) of the representative spin-coated (SC) and slot-die-coated (SD) F8T2 and SPG-01T thin films, calculated using the best-fit parameters derived by SE, (**c**) dependence of thickness on rotational speed and the flow rate for the produced SC and SD samples, respectively, and (**d**) a comparison of the *E_g_^TL^* and E_g_^Tauc^ values for the F8T2 and SPG-01T layers with various thicknesses. The inset depicts the E_g_^Tauc^ value variations for different thicknesses of each polymer and fabrication method.

A 5-TL oscillators dispersion equation was applied for the analysis of the green color-emitting polymers, corresponding to the characteristic interband transitions. Figure 3a,b present the real $\varepsilon_{1}\left(Ε\right)$ and imaginary $\varepsilon_{2}\left(Ε\right)$ part of the calculated dielectric complex $\varepsilon \left(Ε\right)$, using the best-fit parameters derived by the SE data analysis, of each material’s film, representative for both deposition techniques. The spectra clearly demonstrate the presence of sharper and more intense absorption features in F8T2. In comparison with the F8-based copolymer, the absorption edge of SPG-01T is localized to higher energies. Additionally, the energy peaks show consistent behavior for both printed and rigid samples, indicating independence of the optical properties from the fabrication method.

Figure 3c,d depict the films’ thicknesses and band gap values, achieved by applying different rotational speeds and flow rates for the spin-coated and slot-die-coated F8T2 and SPG-01T polymers, respectively. There is an almost linear increase in layer thickness as the first reduces and the latter increases. The produced films of F8T2 are in general thinner, for both techniques, than the corresponding SPG-01T films. This is mainly attributed to the higher molecular weight of the latter, considering that both solutions contain the same concentration of the polymer in common solvent. The determined fundamental band gap (E_g_^TL^) of the primary electronic absorption, which is derived from the TL dispersion equation, is supplemented by additional absorption arising from imperfections, disordering, and nanostructural characteristics like aggregation. By employing Tauc plots, we can calculate the actual band gap (E_g_^Tauc^) excluding the aforementioned additional contributions [33]. For that reason, the latter is spotted at higher energies related to the corresponding E_g_^TL^ values. Generally, higher values of E_g_^Tauc^ are observed for SPG-01T compared with F8T2, independently of the thin film’s thickness. These findings are consistent with the values derived from the absorbance measurements discussed above. However, as shown in the inset of Figure 3d, in the case of SPG-01T, there is a greater variation in the energy gap values corresponding to the different thicknesses whereas comparing the deposition methods, there appears to be larger variations in E_g_^Tauc^ values for the SD coated films. This may arise from the creation of molecule aggregates due to the slower solvent evaporation during the printing process [34]. Table 2 summarizes the results of SE analysis for both polymers.

### 3.3. Dependence of Electroluminescence on Emissive Material’s Thickness

**Figure 4:**
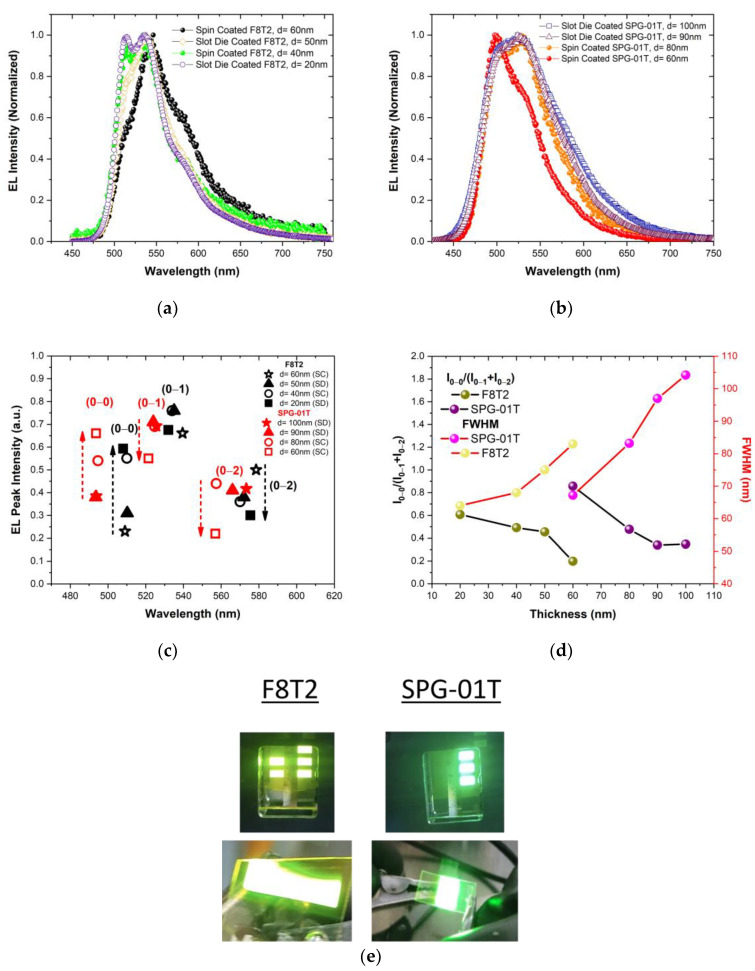
The electroluminescence spectra of the spin-coated and slot-die-coated (**a**) F8T2-based and (**b**) SPG-01T-based PLED devices with sequential thickness decrease. (**c**) The relative intensity of the emission peaks corresponding to different electronic transitions for the sequential thickness decrease (arrows indicate the trend of decreasing thickness). (**d**) The dependence of peak intensity ratios of I_0–0_/(I_0–1_ + I_0–2_) transitions on the thickness and the EL FWHM for the fabricated devices. (**e**) The photos of rigid and flexible PLED devices during operation.

Next, the relationship between the electro-optical characteristics of the fabricated PLED devices with the EML’s thickness was examined. As our major goal is the transition from lab-scale fabrication to in-line production of PLEDs, we sought to demonstrate that the outcomes are independent of the coating process. For this reason, in Figure 4a,b, the EL spectra of functional PLED devices, with sequential EML thickness, fabricated by spin coating and slot-die coating are depicted for F8T2 and SPG-01T, respectively. For both materials, there is a gradual enhancement of (0–0) against the (0–1) and (0–2) transitions because of the arrangement of the polymer’s chains by reducing thickness. This is due to the thin film’s chain packing preference. As has been demonstrated in various organic compounds, there are two favorable intra/interchain structural modulations. J-aggregates are formed when the dominant orientation of the molecules is head to tail, whereas H-aggregates occur when the polymers’ chains are assembled side by side [35]. The J-aggregated molecular behavior is connected with superior optical properties and high orientational order. In H-aggregates, the lowest optical transition is not allowed because neighboring molecules have transition dipoles oriented in opposite directions. This results in a cancellation of the net transition dipole. The preferred nanostructure order can be determined by comparing the relative intensity of the lowest energy (0–0) emission peak to that of the first vibronic (0–1), either at absorbance or emission features [33]. Thus, the higher relative intensity of the (0–0) peak indicates J-aggregates, whereas the opposite means H-aggregates. Recently, a similar thickness-dependent optical behavior for another fluorene-based polymer, F8BT, has been published, and it was attributed to the different π-stacking of fluorene and benzothiadiazole units with J and H aggregate formation going from thinner to thicker layers, respectively [36]. Yuan et al. [37], in their work, concluded that spiro-structure plays a crucial role in influencing intermolecular interactions and the formation of J-aggregated BODIPY dyes. Hence, we can infer that similar structural arrangements are occurring.

In Figure 4c the relative intensities and peak positions of the corresponding (0–0), (0–1), and (0–2) interband transitions are presented and obtained by the deconvolution process of the above EL spectra. As the film thickness of the F8T2 polymer decreases, the intensity of the emission peak at 509 nm (0–0) increases significantly. At the same time, the contribution of the peak at 540 nm (0–1) remains stable, while the contribution of the (0–2) transition peak at 575 nm decreases. When in PLEDs, the SPG-01T is the active layer, and reducing its thickness causes a gradual increase in the (0–0) transition peak at 494 nm. At the same time, the emissions at 524 nm and 599 nm, corresponding to the (0–1) and (0–2) vibronic features, respectively, become weaker.

Color purity of π-conjugated polymers strongly depends on the intrinsic vibronic coupling and structural relaxation of the S_1_ state. Following the Frank–Condon progression, the dominance of the (0–0) transition can be interpreted as a zero offset of equilibrium position between the ground state and excited state [38,39]. For that reason, the ratio of the peak intensities of the (0–0), (0–1), and (0–2) EL transitions I_0–0_/(I_0–1_ + I_0–2_) is introduced. As it is presented in Figure 4d separately for each polymer, the higher values the fraction approaches, the larger orbital overlap occurs, resulting in narrower emission with lower FWHM. Therefore, the changes of structural configuration in the two polymers imposed by thickness reduction lead to the progressive increment of the fraction’s values and, consequently, the narrowband emission of F8T2 and SPG-01T with FWHM 64 nm and 67.2 nm for the thinner films, respectively. It can be concluded that F8T2, which exhibits more intense spectral shifts with thickness decrease, has a higher tendency to the formation of J-aggregates. The transition between H and J-aggregates can be initiated by a longitudinal displacement of molecules relative to each other, which closely mirrors the chemical structure of the involved chromophores. Conceivably, this propensity of F8T2 for the J-aggregates mode is related to the presence of the two alkyl chains connecting to the bridging benzylic position of the molecule that results in a significant intermolecular distance of at least 4.5 Å while, in the case of SPG-01T, the morphological stability raised by the spiro junction between the two molecular halves hardens the structural modifications [40,41,42]. In Figure 4e, photos of rigid and flexible PLEDs based on both emitting materials during operation are presented.

### 3.4. Electrical Performance of the Fabricated PLEDs

**Figure 5:**
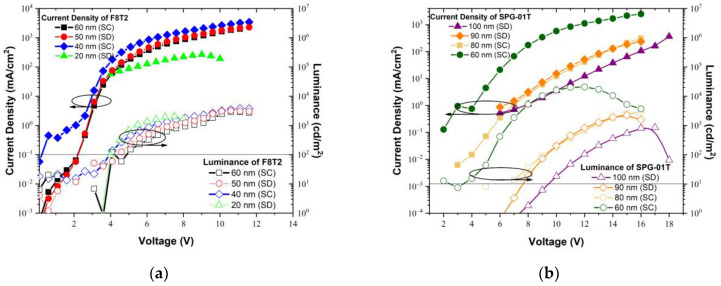
Current density-voltage (J-V) and luminance-voltage (L-V) characteristic curves of the selected slot-die-coated (SD) and spin-coated (SC) PLEDs with sequential thicknesses of (**a**) F8T2 and (**b**) SPG-01T.

Moving on to the operational characteristics of the fabricated PLEDs based on the F8T2 and SPG-01T polymers, the current density (J) and luminance (L) versus bias voltage (V) diagrams are presented in Figure 5a,b correspondingly for the different EMLs’ thicknesses of the spin-coated and printed devices. For each emissive material, in a fixed device architecture, there is an optimal relationship of electro-optical properties with the layer’s thickness. Thinner films favor higher current densities while thicker layers suffer from poor light extraction efficiencies due to photons coupled to waveguide modes within the device [43]. In our investigation, moving from thicker to thinner films, independently of the material and the fabrication method, we can note the expected gradual increase of the operational current density. However, in the case of F8T2, the printed device with the thinner EML layer at 20 nm does not follow this trend, probably due to its poor lifetime because of the excessive Joule heating. Quenching effects and coupling to the cathode (plasmons) become more pronounced in ultra-thin layers, as electrons and holes recombine closer to the electrode. In addition, such non-radiative recombination processes can lead to increased heat generation and consequently limit the PLED’s lifetime [44]. Basically, the devices that have the F8T2 as an active layer operate with higher J values by both fabrication methods, even at a comparable thicknesses with the SPG-01T diodes. Considering the optical performance of the rigid light-emitting diodes for SPG-01T, the highest luminance efficiency is 20,690 cd/m^2^ with an EML thickness of 60 nm, whereas, for F8T2, the maximum luminance achieved is 3183 cd/m^2^ with a 50 nm active layer thickness. Comparing the functional diodes with a similar thickness of the emissive layer at 60 nm and the same fabrication technique, the luminance values of SPG-01T-based PLEDs are significantly higher than the corresponding values of F8T2-based devices. This can be justified by the more proper alignment of the SPG-01T’s energy levels with the HOMO-LUMO levels of the neighboring layers of our architecture discussed above as the efficiency of light output strongly depends on the balanced injection of holes and electrons in the recombination zone [45]. The L-V curves of F8T2 demonstrate significant fluctuations at low voltages due to leakage currents, likely due to the low thickness of the produced films, which increases the likelihood of voids and defects forming. Therefore, the turn-on voltage (V_on_) of the corresponding PLEDs was defined at 100 cd/m^2^. By lowering the thickness of F8T2, the V_on_ is reduced from 4.9 V to 3.9 V. SPG-01T-based PLEDs demonstrate similar behavior, with the V_on_ (at 10 cd/m^2^) decreasing from 9.2 V to 3 V as the film thickness decreases. The wider variation of the latter could be attributed to the thicker layers achieved. Table 3 summarizes the numerical results of the electrical characteristics of the studied PLEDs as well as the FWHM values of the EL emission spectra.

**Figure 6:**
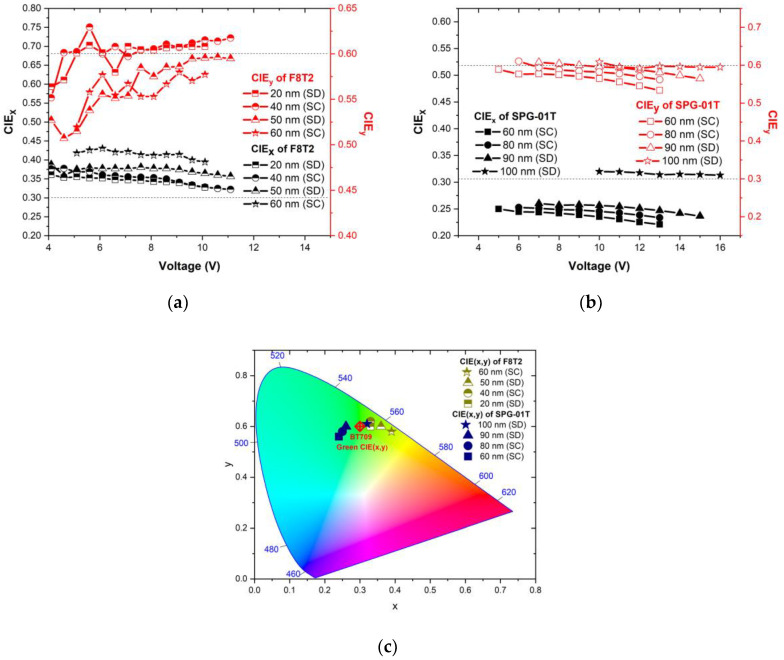
The evolution of EL Chromaticity Coordinates of the selected SD and SC PLEDs with sequential thicknesses of (**a**) F8T2 and (**b**) SPG-01T by increasing voltage. The horizontal dotted lines denote the respective (x, y) coordinates of BT709 recommended standard green CIE coordinates. (**c**) The chromaticity coordinates at 10 V operational voltage of the produced F8T2 and SPG-01T PLEDs.

Figure 6a,b show the evolution of the CIE coordinates with an increasing operational voltage of the rigid and flexible devices with various EML thicknesses. Basically, the CIE_y_ of F8T2 exhibits intensive deviation by increasing bias voltage while the CIE_x_ remains relatively stable. A very interesting fact is that, by implementing thinner emissive layers of F8T2, the values of x coordinates are reduced and simultaneously the y coordinates are increased in such a way that color emitting by the thinnest film, having the narrower emission as discussed in the previous section, approaches the ideal CIE coordinates set by BT709. On the other hand, by lowering the thickness of the SPG-01T active layers, the EL color coordinates move away from the ideal values. However, PLEDs containing SPG-01T as an emissive polymer present remarkable color stability even at higher luminance levels. Therefore, PLEDs containing a thin EML of F8T2 are more promising candidates for wearable lighting. Conversely, increased thickness enhances the devices composed of SPG-01T as the active layer.

### 3.5. Detection of Rhodamine 6G

**Figure 7:**
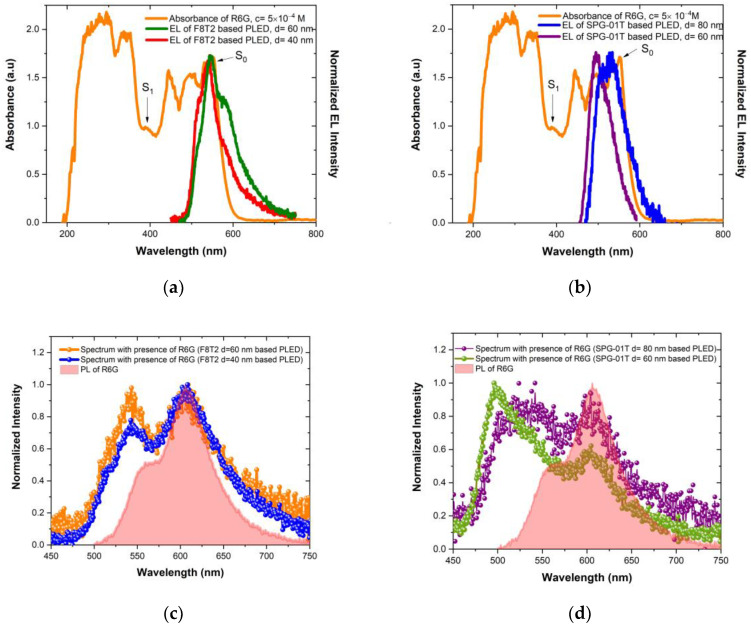
The UV-Vis absorption of R6G and the electroluminescence spectra of (**a**) F8T2 and (**b**) SPG-01T-based spin-coated PLEDs with different thicknesses of EMLs. The PL spectrum of R6G and the measured spectrum after its illumination by the (**c**) F8T2 and (**d**) SPG-01T-based spin-coated PLEDs with different thicknesses of EMLs.

Finally, this study explored the PLEDs’ ability to detect Rhodamine 6G dye using both polymers. We aimed to investigate the dependence of the optical detection of R6G’s PL on the spectral characteristics of the two green color-emitting polymers and their thicknesses when implemented in PLEDs as an emissive layer. For that reason, we used, for convenience, the spin-coated devices. In Figure 7a,b the absorbance of 5 × 10^−4^ M R6G is depicted comparatively with the EL emission spectra of F8T2 and SPG-01T, respectively. For the R6G, the monomer and aggregates contributions caused a broadband spectrum in the range 432–584 nm and the peak around 530 nm is attributed to S_0_ → S_1_ excitation. The rest of the observed peaks at 387, 347, 276, and 247 nm correspond to the excited singlets S_1_, S_2_, S_3_, and S_4,_ respectively [46]. Additionally, in both Figure 7c,d the PL spectrum of R6G is presented as having a maximum at 606 nm and a vibronic shoulder at 558 nm, similarly as reported elsewhere [47]. A more selective and accurate overlapping with the R6G’s S_0_ absorption peak is observed in the case of the F8T2 EL emission for both thicknesses in comparison with the broader and blue-shifted EL emission of the corresponding SPG-01T-based devices. By reducing the thickness of the F8T2 emissive layer, the peak of the EL emission is shifted to lower wavelengths compared to the 530 nm absorption peak. This shift is also accompanied by a decrease in FWHM, which improves the alignment between R6G absorption and F8T2 emission. A shift to lower wavelengths and a decrease in the FWHM is also observed in the case of the SPG-01T with the decrease in thickness, but the match between the absorption peak and the λ emission is not favored. Then we measured the signal derived from the PLED devices’ emissions, with the presence of R6G in a cuvette, inside an integrating sphere. The recorded spectra are plotted comparatively to PL emission of R6G in Figure 7c,d for F8T2 and SPG-01T, respectively. The optical sensing of the PL emission peak of R6G at 606 nm is easily extracted using both polymer-based PLEDs. As expected from the above results, in the case of SPG-01T, the detected peak exhibits lower intensity with higher noise. On the other hand, in the emission spectrum of F8T2-based PLEDs, the PL signal of R6G is clearly distinguished. Concerning thickness, when transitioning from thicker to thinner emissive films for F8T2, the spectrum shows only a slight decrease in noise. However, in the case of SPG-01T, the intensity of the R6G peak is even weaker. We can assume that this is due to the less drastic relocation of F8T2’s EL spectrum compared to the EL emission of SPG-01T when the thickness is lowered, relative to the S_0_ absorption peak of R6G. Additionally, it should be mentioned that in both polymers there is contribution of the PLED’s emission in the measured signal. At this point, it could be highlighted that this study is very important as a step forward for the application of organic light-emitting diodes in the field of wearable technology. By enhancing the understanding and potential of OLEDs, this research holds the promise of enabling new diagnostic and therapeutic techniques, thereby opening new avenues for their integration into biomedical applications [48,49].

## 4. Conclusions

In summary, we explored the potential of two green color-emitting polymers, F8T2 and SPG-01T, as emissive layers in PLEDs for wearable applications. We focused on operational stability, spectral selectivity, and operational efficiency. Functional rigid and flexible devices bearing the two polymers exhibited high luminance efficiencies. Thus, the printability and up-scalability of both polymers were proved, paving the way toward large-scale R2R PLED production. Furthermore, the detailed exploration of the device architecture, particularly the influence of varying emissive layer thicknesses, provided valuable insights into the aggregate behaviors of F8T2 and SPG-01T green color emitting polymers. This aggregate behavior plays a critical role in determining device performance, particularly in terms of EL emission efficiency, color purity, and stability. Given the growing demand for more efficient PLEDs in wearable lighting and current trends such as optical sensing applications, the universality of our results, independent of the fabrication process, was a significant finding.

By reducing the thickness of the F8T2 active layer in PLEDs, green color with a higher purity is emitted sacrificing, however, operational properties due to the high current density developed. On the contrary, increasing the thickness of SPG-01T brings the color coordinates closer to the ideal National Television Union standards. However, this also leads to a deterioration in electrical properties due to the increased density of charge carrier traps at the thick layer. Consequently, there is a need for further investigation of alternative architectures in order to achieve a balance between optical and electrical performance for wearable lighting applications.

Concerning the optical detection of R6G’s photoluminescence, the PLED’s spectral characteristics and the EML’s thickness were found to be significant criteria for the selection of the proper emitting polymer when applied in a PLED-based optical sensor. From the preliminary results, it is derived that F8T2-based PLEDs operate better as actuators, probably due to the most vibronic spectral characteristics and selective overlap with the fluorescent dye’s absorption S_0_ peak compared to the broader corresponding emission of SPG-01T-based PLEDs. When the thickness is reduced, the electroluminescence of SPG-01T shifts towards the blue, resulting in a lower intensity and higher noise in the detected signal. On the other hand, the electroluminescence of F8T2 remains relatively stable due to a less intense shift in the emission peak compared to the S_0_ absorption maximum of R6G. In conclusion, F8T2 layered PLEDs can be used in wearable biosensing platforms with specific selectivity requirements. On the other hand, light-emitting devices with SPG-01T may be more suitable for optical detection across a wider range of wavelengths.

## Figures and Tables

**Table 1:** Summary of photophysical and electrochemical properties of F8T2 and SPG-01T.

Polymer	Photophysical Properties				Electrochemical Properties		
	λ_Abs_ (nm)	λ_onset_ (nm)	E_g_^Opt^ (eV)	λ_PL_ (nm)	E_onset_ (V)	E_HOMO_ (eV)	E_LUMO_ (eV)
F8T2	453 (0–1), 484 (0–0)	519	2.39	510 (0–0), 542 (0–1), 581 (0–2)	1.23	−5.6	−3.2
SPG-01T	343 (0–3), 367 (0–2), 397 (0–1), 442 (0–0)	490	2.53	509 (0–0), 537 (0–1)	0.91	−5.2	−2.7

**Table 2:** SE analysis of rigid and flexible F8T2 and SPG-01T samples with different thicknesses, fabricated by spin coating and slot-die coating, respectively.

Polymer	Fabrication Method							
	Spin Coating				Slot-Die Coating			
	Speed (rpm)	Thickness (nm)	E_g_^TL^ (eV)	E_g_^Tauc^ (eV)	Flow Rate (mL/min)	Thickness (nm)	E_g_^TL^ (eV)	E_g_^Tauc^ (eV)
F8T2	1000	58.8	2.40	2.4972	0.03	20.5	2.27	2.45
	2000	49.4	2.39	2.4933	0.035	21.4	2.27	2.46
	3000	40.7	2.39	2.4856	0.04	24.2	2.25	2.45
					0.05	33.3	2.29	2.44
					0.06	40.9	2.31	2.43
					0.08	48.1	2.31	2.42
SPG-01T	1000	83.2	2.50	2.6508	0.04	57.8	1.81	2.78
	2000	66.1	2.60	2.6778	0.06	69.2	1.90	2.80
	3000	57.6	2.57	2.6713	0.08	78.4	1.92	2.70
					0.10	91.8	1.58	2.74
					0.11	98.1	1.36	2.70

**Table 3:** Dependence of spectral (FWHM) and electrical characteristics on emissive layer thickness.

Polymer	Thickness (Fabrication Method) (nm)	Electrical Characteristics			
		FWHM (nm)	^1^ V_on_ (V)	Max. Luminance (cd/m^2^)	^2^ V_op_ (V)
F8T2	60 (SC)	83	4.9	2907	4.7
	50 (SD)	75	4.6	3183	4.4
	40 (SC)	68	4.1	1963	3.8
	20 (SD)	64	3.9	2023	5.3
SPG-01T	100 (SD)	104.2	9.2	854	16
	90 (SD)	97	7.5	2432	13.6
	80 (SC)	83.2	7	2808	13.5
	60 (SC)	67.2	5	20,690	7.4

^1^ V_on_: Turn-on Voltage at 100 cd/m^2^ for F8T2 and at 10 cd/m^2^ for SPG-01T, ^2^ V_op_: Operation Voltage with 100 mA/cm^2.^

## Data Availability

The datasets utilized and/or examined in this study can be obtained from the corresponding author upon making a reasonable request.
